# Three-dimensional analysis of the characteristics of joint motion and gait pattern in a rodent model following spinal nerve ligation

**DOI:** 10.1186/s12938-021-00892-6

**Published:** 2021-06-05

**Authors:** Takayuki Seto, Hidenori Suzuki, Tomoya Okazaki, Yasuaki Imajo, Norihiro Nishida, Masahiro Funaba, Tsukasa Kanchiku, Toshihiko Taguchi, Takashi Sakai

**Affiliations:** 1 Department of Orthopaedic Surgery, Yamaguchi University Graduate School of Medicine, 1-1-1 Minamikogushi, Ube, Yamaguchi 755-8505 Japan; 2 Department of Spine and Spinal Cord Surgery, Yamaguchi Rosai Hospital, Sanyoonoda, Yamaguchi Japan; 3 Department of Orthopaedic Surgery, Yamaguchi Rosai Hospital, Sanyoonoda, Yamaguchi Japan

**Keywords:** Three-dimensional gait analysis, Sciatic nerve ligation, Three-dimensional kinematic analysis, Functional evaluation

## Abstract

**Background:**

The spinal nerve ligation (SNL) rat is well known as the most common rodent model of neuropathic pain without motor deficit. Researchers have performed analyses using only the von Frey and thermal withdrawal tests to evaluate pain intensity in the rat experimental model. However, these test are completely different from the neurological examinations performed clinically. We think that several behavioral reactions must be observed following SNL because the patients with neuropathic pain usually have impaired coordination of the motions of the right–left limbs and right–left joint motion differences. In this study, we attempted to clarify the pain behavioral reactions in SNL rat model as in patients. We used the Kinema-Tracer system for 3D kinematics gait analysis to identify new characteristic parameters of each joint movement and gait pattern.

**Results:**

The effect of SNL on mechanical allodynia was a 47 ± 6.1% decrease in the withdrawal threshold during 1–8 weeks post-operation. Sagittal trajectories of the hip, knee and ankle markers in SNL rats showed a large sagittal fluctuation of each joint while walking. Top minus bottom height of the left hip and knee that represents instability during walking was significantly larger in the SNL than sham rats. Both-foot contact time, which is one of the gait characteristics, was significantly longer in the SNL versus sham rats: 1.9 ± 0.15 s vs. 1.03 ± 0.15 s at 4 weeks post-operation (*p* = 0.003). We also examined the circular phase time to evaluate coordination of the right and left hind-limbs. The ratio of the right/left circular time was 1.0 ± 0.08 in the sham rats and 0.62 ± 0.15 in the SNL rats at 4 weeks post-operation.

**Conclusions:**

We revealed new quantitative parameters in an SNL rat model that are directly relevant to the neurological symptoms in patients with neuropathic pain, in whom the von Frey and thermal withdrawal tests are not used at all clinically. This new 3D analysis system can contribute to the analysis of pain intensity of SNL rats in detail similar to human patients’ reactions following neuropathic pain.

**Supplementary Information:**

The online version contains supplementary material available at 10.1186/s12938-021-00892-6.

## Background

The spinal nerve ligation (SNL) model has been widely adopted and is frequently used within various functional evaluation studies of neuropathic pain [1–4]. Pain cannot be directly measured in animals; instead, the intensity of pain is inferred from “pain-like” behaviors, such as withdrawal from a nociceptive stimulus, which is the most commonly used method to quantify nociception in animal studies [5–8]. Researchers have performed analyses using only von Frey and thermal withdrawal tests to evaluate pain intensity in the SNL model, but these tests are completely different from clinical neurological examinations used in humans [5]. Spontaneous withdrawal reactions are strongly affected by spinal reflex, and scientific evidence that the reactions are a specific reflex against pain is lacking [5]. In addition, the von Frey withdrawal test has some issues of test–retest reliability and inter-rater reliability [5, 9–14]. The test cannot reveal impaired coordination of the motions of the right–left lower limbs in rodent models following SNL or spinal cord injury [15–23]. A previous report raised issues about preclinical studies in pain research especially in regard to the methodology of pain monitoring. Animal experiments have produced an explosion of information about pain, but this knowledge has failed to yield new painkillers for use in humans. This abysmal track record has led to calls to overhaul how pain intensity should be monitored in preclinical studies [5]. For these reasons, new evaluation methods are desired when conducting research using the SNL model [5, 16, 17].

We think that several behavioral reactions must be observed following SNL because patients with neuropathic pain usually have impaired coordination of the motions of the right–left limbs and differences in right–left joint motion. In this study, we attempted to clarify the pain behavioral reactions similar to those in patients also in a SNL rat model. Several approaches were reported using a 2-dimensional (2D) catwalk system and 3-dimensional (3D) treadmill analysis systems. Previous gait analysis indicated significant differences for foot print areas, maximum contact maximum intensity, stand phase, swing phase, single stance and regular index with sham and/or SNL model comparisons [12, 13]. However, the objective parameters available to pain response in the SNL model have not been shown in the previous reports.

We attempted to elucidate the possibility of 3D gait analysis of the SNL model that could reveal very slight gait loss even though a previous paper reported that the SNL model showed no motor deficit based on conventional evaluations [4]. We sought to establish standardized 3D parameters that can be generally used for research with the SNL rat model. In this study, we represented the amount of flail in each joint numerically by calculating the top minus bottom height of each joint on the side of SNL. In addition, we represented walking disability by measuring the differences of step length, stride length, both-foot contact time and circular phase between the right and left lower limbs in the SNL model. We think these new parameters can also be applied to patients with sciatic nerve disorder following treatment and could become the standard scoring system for sciatic nerve function in gait deficit analysis.

## Results

### Mechanical allodynia

**Fig. 1:**
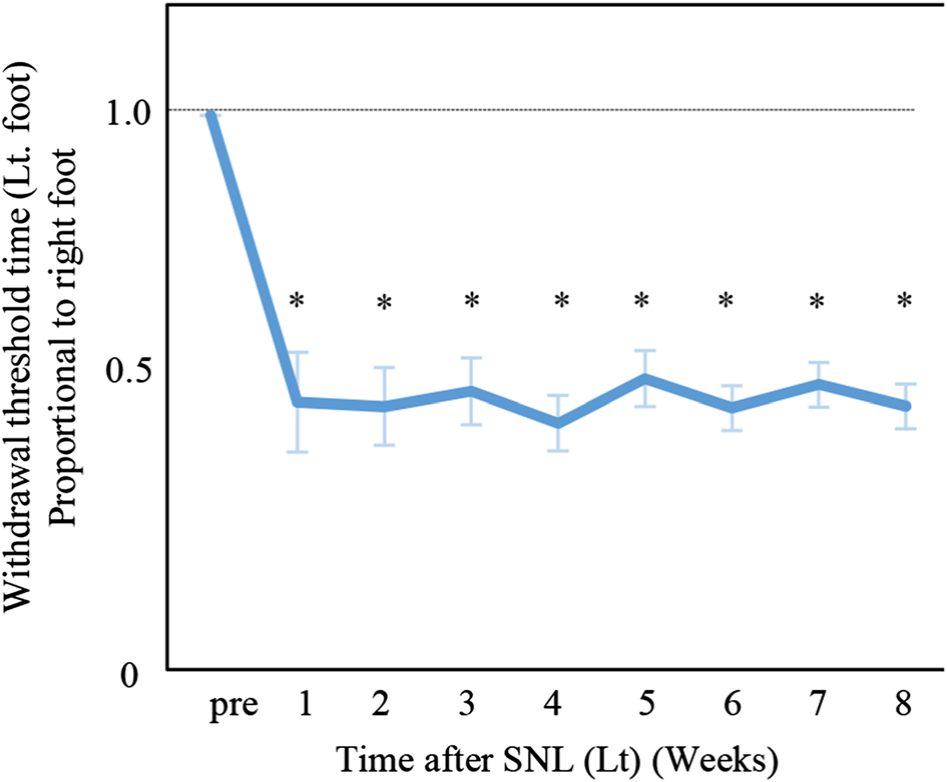
Tactile paw withdrawal threshold time (Lt. foot) proportional to right foot in SNL rats. The effect of SNL on mechanical allodynia was a 47 ± 6.1% decrease in the withdrawal threshold following left sciatic nerve damage. Significant differences in the left foot compared to the right foot were observed at all time points. ^*^p  < 0.01

The effect of SNL on mechanical allodynia was a 47 ± 6.1% (95% confidence interval, 43–51%) decrease in the withdrawal threshold for the operated left hind paw compared to the right normal hind paw during 1–8 weeks post-operation (Fig. 1). Significant differences in withdrawal threshold time between left and right hind paws were observed by paired *t-*test at all time points from 1 to 8 weeks (*p* < 0.01 for all comparisons).

### Three-dimensional kinematic analysis

Sagittal trajectories of the hip, knee and ankle markers in SNL rats were analyzed at pre-operation and 1, 2 and 4 weeks after left SNL. We show the data at 1 and 4 weeks after SNL and of the sham rats in Figs. 4, 5 and 6. These joints on the SNL side were unstable compared to those on the right side. We measured the sagittal fluctuations of each joint in walking to calculate the top minus bottom height of each marker. The trajectories indicate the vertical joint movement during the stance and swing phase of gait. We illustrate the meaning of the stance and swing phase in walking in Additional file 1: Fig S1.

### Sagittal trajectories of the hip joint

**Fig. 2:**
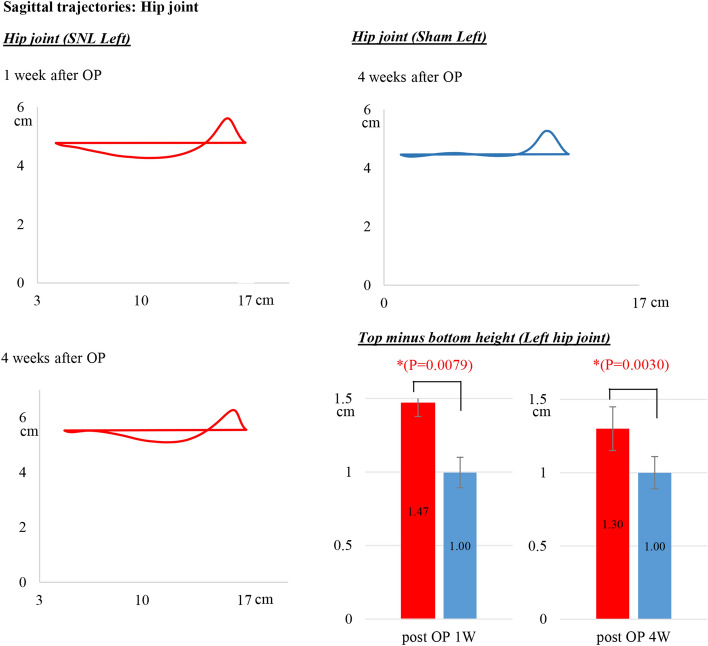
Sagittal trajectories of the hip joint by 3D kinematics gait analysis. Top minus bottom height of the left hip marker, indicating the fluctuation of the hip joint, during walking in SNL rats was 1.47 ± 0.33 cm at 1 weeks post-operation and 1.30 ± 0.25 cm at 4 weeks post-operation. The values of the sham left hind-limbs were 1.47 vs. 1.0 cm and 1.30 vs. 1.0 cm, respectively. OP, operation

The motion of the sham rat’s hip joint (blue line in the top right diagram of Fig. 2) moved linearly in a horizontal direction during the stance phase and swung up a little vertically in an arc during the swing phase and forward movement. The trajectory of the hip joint in sham rats during the stance phase was a flat line and the last 80% of the swing phase was also a flat line (Fig. 2). However, 1 week after SNL of the left hind-limb, the trajectories of the left hip joint tended to move downward following upward movement during the swing phase (red line in the top left diagram of Fig. 2), indicating left hip instability. Four weeks after SNL, the tendency to move downward was decreased during the swing phase. The top minus bottom height of the left hip marker, which indicates the fluctuations of the hip joint during walking in SNL rats was 1.47 ± 0.33 cm at 1 weeks post-operation and 1.30 ± 0.25 cm at 4 weeks post-operation. Compared to sham left hind-limbs, 1.47 vs. 1.0 cm and 1.30 vs. 1.0 cm, respectively, both fluctuations were significantly different (*p* < 0.01).

### Sagittal trajectories of the knee joint

**Fig. 3:**
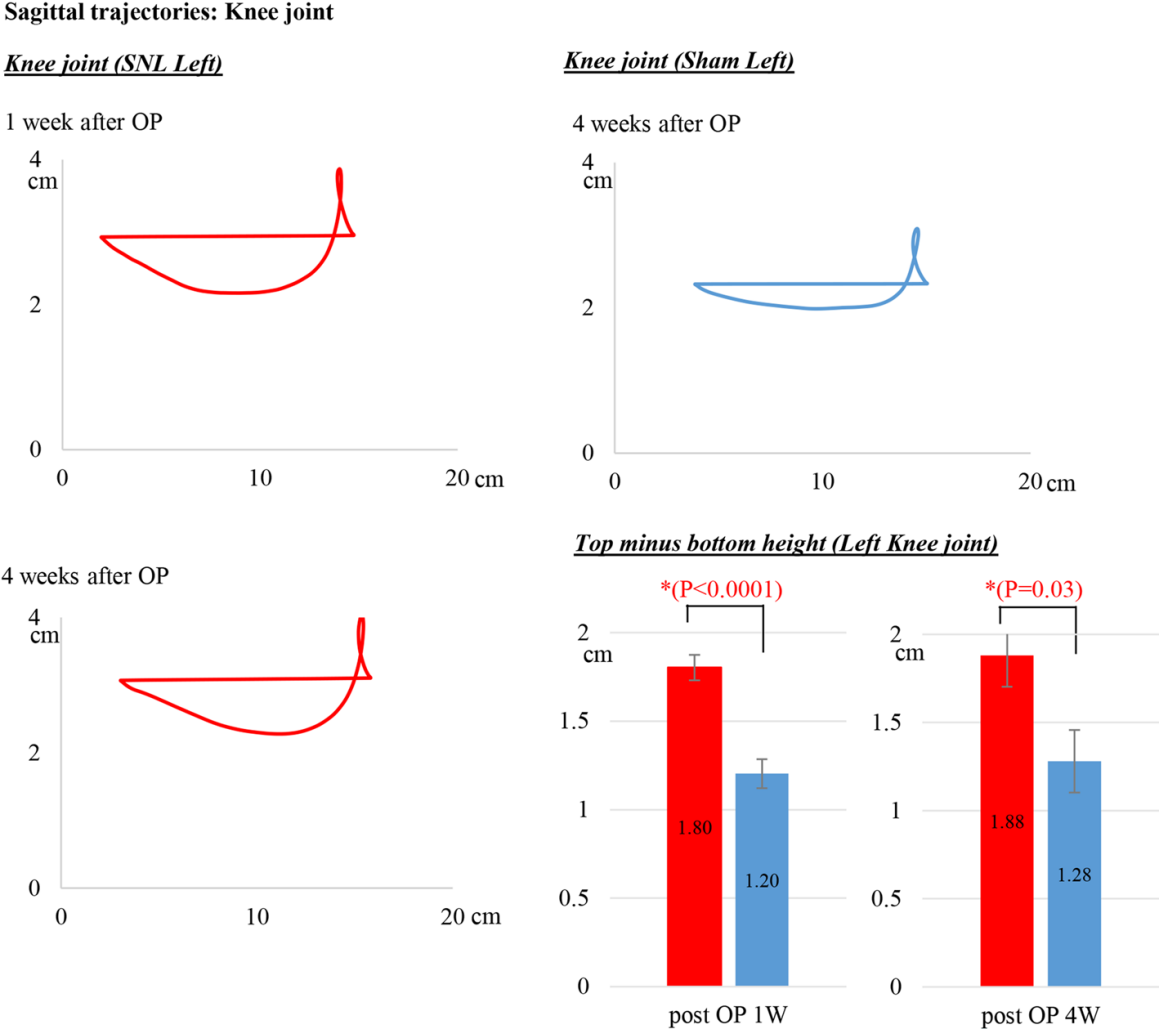
Sagittal trajectories of the knee joint by 3D kinematics gait analysis. Top minus bottom height of the left knee marker, indicating fluctuations of the knee joint, during walking in the SNL rats was 1.80 ± 0.32 cm at 1 weeks post-operation and 1.88 ± 0.45 cm at 4 weeks post-operation. These values were significantly different compared with those of the sham left hind-limbs: 1.80 vs. 1.20 cm and 1.88 vs. 1.28 cm, respectively. ^*^p < 0.0001 at 1 weeks post-operation and *p* = 0.03 at 4 weeks post-operation. OP, operation

The motion of the sham rat’s knee joint (blue line in the top right diagram of Fig. 3) moved similarly to that of the hip joint. However, the knee marker was not flat during the swing phase. In SNL, movement more downward during the swing phase (red line, left diagrams in Fig. 3) indicates left knee instability. The top minus bottom heights of the left knee marker, which indicates the fluctuations of the knee joint during walking in SNL rats were 1.80 ± 0.32 cm at 1 weeks post-operation and 1.88 ± 0.45 cm at 4 weeks post-operation and were significantly different compared with those of the sham left hind-limbs, 1.80 vs. 1.20 cm and 1.88 vs. 1.28 cm, respectively (Fig. 3). Both fluctuations were significantly different (*p* < 0.05).

### Sagittal trajectories of the ankle joint

**Fig. 4:**
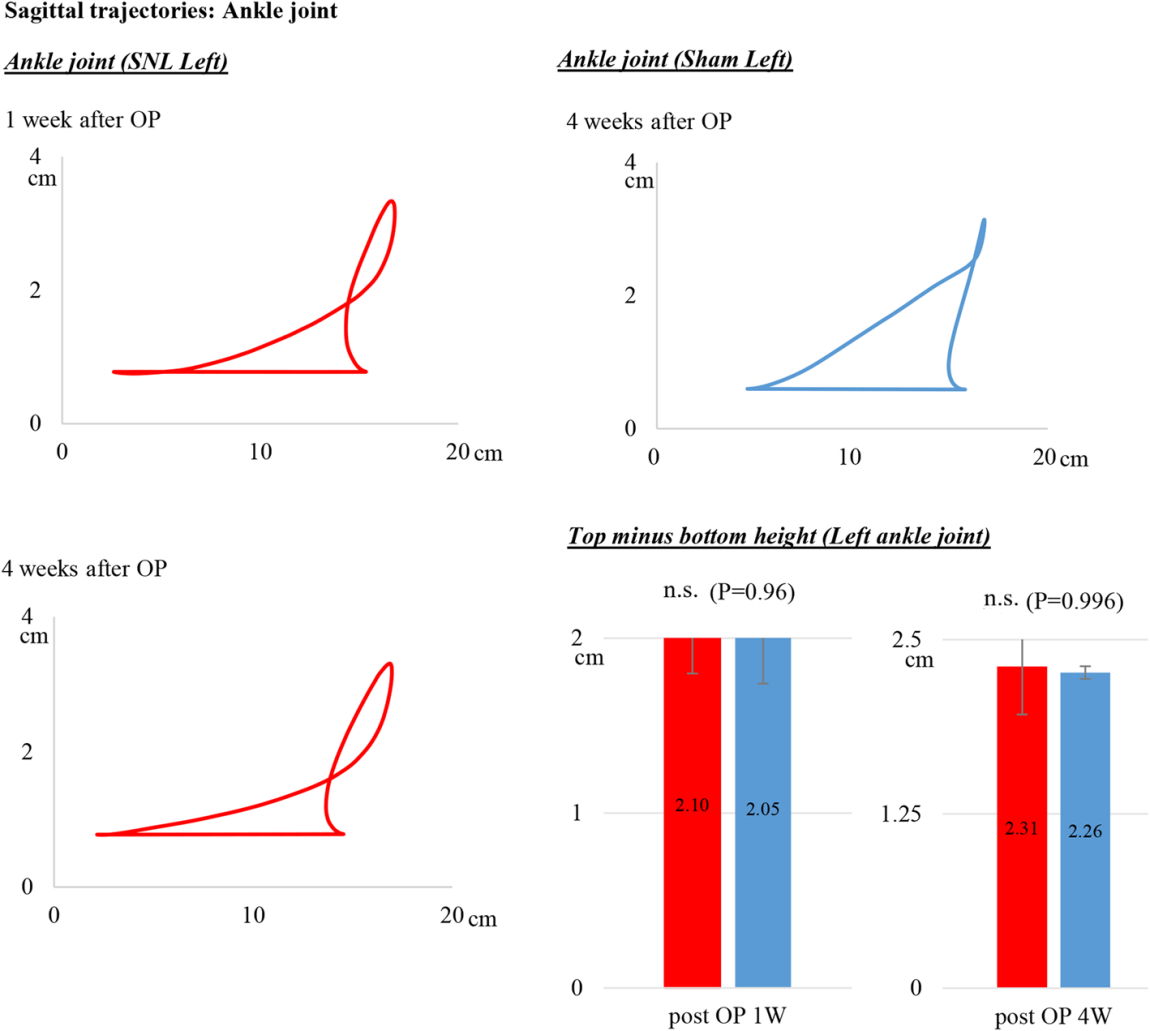
Sagittal trajectories of ankle joint by 3D kinematics gait analysis. The angle of rise during the first 2/3 of the swing phase in SNL, left diagrams, was smaller than that in the sham. The SNL rats continuously moved the ankle up more rapidly during the last 1/3 of the swing phase compared to that in the sham rats. These trajectory patterns defined the characteristic ankle movement in SNL rats. OP, operation; n.s

The ankle joint in the sham rats (blue line in the top right diagram of Fig. 4) gradually rose during the stance to swing phases. In contrast, the ankle joint in SNL rats rose sharply up and down between consecutive swing phases (red line, left diagrams in Fig. 4). The top minus bottom height of the ankle marker during walking in the SNL rats was not different compared with that in the sham rats. However, the angle of rise during the first 2/3 of the swing phase was smaller than that of the sham rats. The SNL rats continuously moved the ankle up more rapidly during the last 1/3 of the swing phase compared to the action in the sham rats. These trajectory patterns defined the characteristic ankle movement in SNL rats (Fig. 4).

### Gait characteristics following SNL

**Fig. 5:**
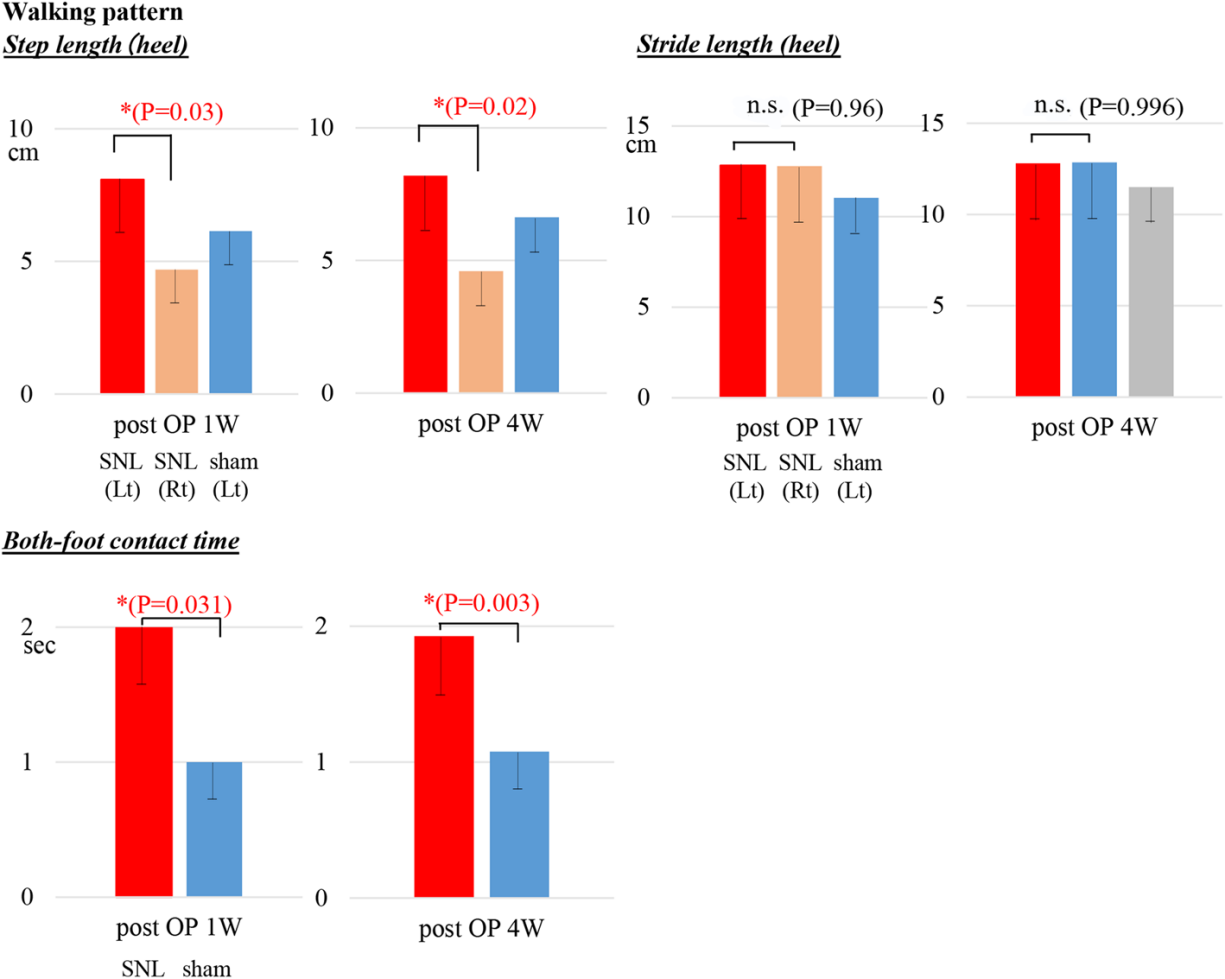
Walking characteristics of step length (heel), stride length (heel) and both-foot contact time. Heel step length was significantly longer for the left compared to right side at 1 weeks and 4 weeks post-operation in the SNL rats. However, heel stride length did not differ between the left and right limbs in the SNL rats. Both-foot contact time was significantly longer in the SNL rats compared to the sham. ^*^p < 0.05; SNL model, *n* = 10; sham model, *n* = 10. n.s

We analyzed the gait characteristics following SNL compared to sham rats or the right hind-limb at 1 weeks and 4 weeks after operation. The heel stride length (Additional file 1: Fig S1) did not differ between the left and right limbs in the SNL rats: left limb, 12.8 ± 0.9 cm, right limb, 12.8 ± 0.3 cm at 1 weeks and 12.7 ± 0.7 cm and 12.9 ± 0.2 cm, respectively, at 4 weeks (Fig. 5).

Heel step length (Additional file 1: Fig S1) was significantly longer on the left compared to the right side at 1 weeks and 4 weeks post-operation in the SNL rats (*p* < 0.05) (Fig. 5). Step lengths were 8.1 ± 0.9 cm on the left and 4.7 ± 0.3 cm on the right at 1 weeks and 8.2 ± 0.7 cm on the left and 4.6 ± 0.2 cm on the right at 4 weeks.

Both-foot contact time is the overlap time of both feet in the stance phase during walking. Both-foot contact time was significantly longer in the SNL rats compared to the sham: 2.0 ± 0.25 s in the SNL and 1.0 ± 0.26 s in the sham at 1 weeks (*p* < 0.05) and 1.9 ± 0.15 s and 1.03 ± 0.15 s, respectively, at 4 weeks (< 0.001) (Fig. 5).

### Circular gait pattern following SNL

**Fig. 6:**
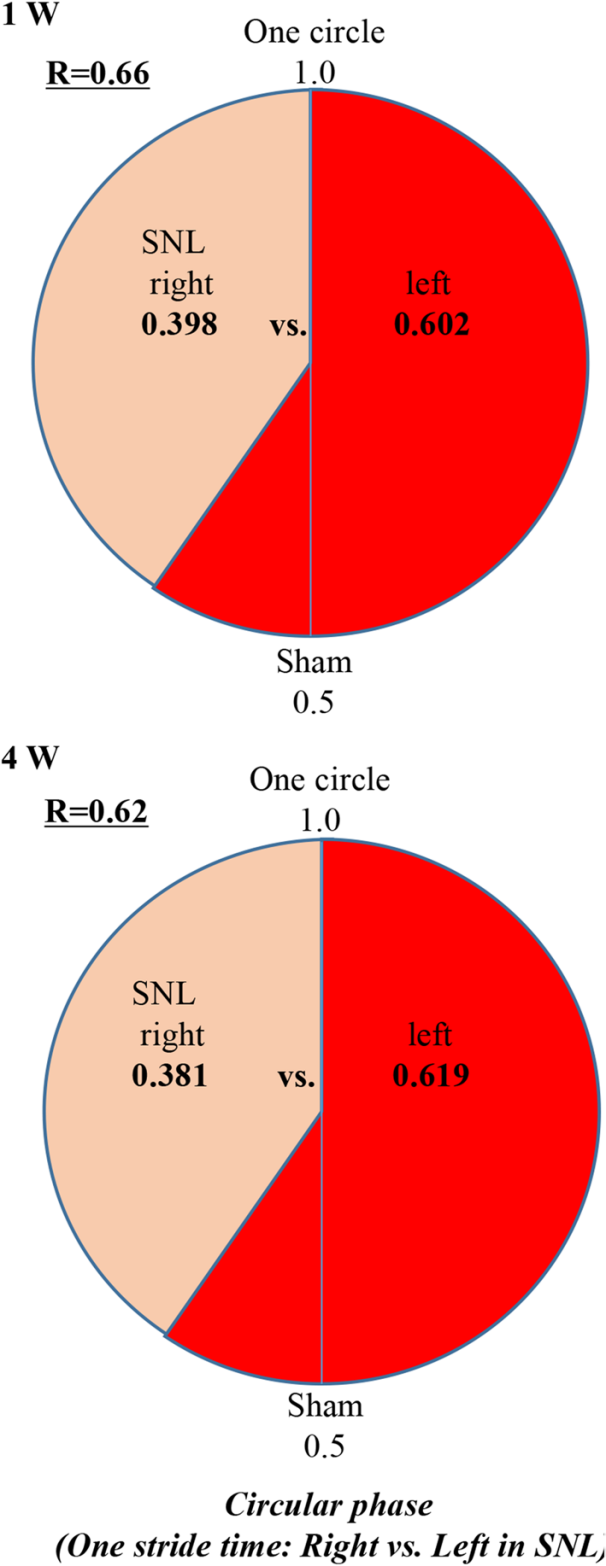
Gait characteristics of SNL rats in circular phase. The touchdown phases of both hind-limbs were quantitatively assessed within one cycle as the time taken from when the right hind-limb lifted off and touched down again. The R value is the ratio of the right/left circular time. The *R* values were 1.0 ± 0.08 in the sham rats and 0.66 ± 0.20 at 1 weeks and 0.62 ± 0.15 at 4 weeks post-operation in the SNL rats. SNL model, *n* = 10; sham model, *n* = 10

We also examined the circular phase time in each hind-limb to evaluate the coordination of the right and left hind-limbs during walking [16, 17]. The analysis provided touchdown and lift-off events during multiple gait cycles. The *R* value is the ratio of the right/left circular time. In the circular phase, the *R* values were 1.0 ± 0.08 in the sham rats and 0.66 ± 0.20 at 1 weeks and 0.62 ± 0.15 at 4 weeks in the SNL rats (Fig. 6).

## Discussion

Since its introduction in 1992, the SNL model of neuropathic pain has been widely used for various investigative works on neuropathic pain mechanisms and in screening tests for the development of new analgesic drugs [1–4].

Researchers using the SNL model have traditionally relied upon simple reflex tests such as the paw withdrawal test to assess neuropathic pain because the SNL model does not show any motor deficit [4]. However, such responses to evoked pain do not closely match the experience of continuous pain because they detect only spinal reflex and not the excitation by filament stimulation [2, 3, 8, 9, 12]. The SNL model basically has no motor deficit [3, 4]. These methods are therefore unsuitable for the clinical assessment of allodynia and chronic pain because they do not evaluate the animal’s pain behavior [5]. Gait analysis is promising for the evaluation of neurological deficits as gait is a fundamental, physiological and unforced form of locomotion with direct clinical relevance. 3D digital gait analysis could be a useful tool for detecting the severity of neuropathic pain and the change in gait characteristics following SNL [11, 14–24]. Indeed, some authors claim that 2D catwalk analysis is useful for assessing animal pain [9, 10, 12, 13], and these types of measures are relatively easy to assess [13, 21].

Quantitative measures available from 3D gait analysis include phase lag, symmetry, complexity and range of motion [16–18, 23, 24]. Left–right symmetry is perhaps the most easily identifiable measurement. Uninjured individuals tend to have gait patterns that exhibit left–right symmetry [16, 17]. Moreover, subtle changes in walking rhythm and other detailed patterns can be revealed by the Kinema-Tracer system with 3D dimensional data following injury [16, 17]. Previous papers revealed the improved toe clearance and touchdown phase of both hind-limbs following treatment in the field of spinal cord injury [15–17]. In addition, several papers showed the recovery of gait pattern objectively using 3D gait analysis following the treatment of neurodegenerative disorders [18, 24].

In the present study, we provided several new parameters in 3D gait analysis of an SNL model: the sagittal trajectories of each joint and the vertical instability, the heel step length, the both-foot contact time and the circular phase. First, we showed the sagittal trajectories of hind-limb joints and the characteristics of SNL hind-limb motion. The 3D dimensional data quantified the slight vertical fluctuations and the characteristic trajectories of each joint in walking. The top minus bottom height of the left hip marker was 1.47 ± 0.33 cm at 1 week post-operation and 1.30 ± 0.25 cm at 4 weeks post-operation. Compared to sham left hind-limbs, 1.47 vs. 1.0 cm and 1.30 vs. 1.0 cm, respectively, both fluctuations were significantly different. Similarly, the left knee marker values in the SNL rats were 1.80 ± 0.32 cm at 1 week post-operation and 1.88 ± 0.45 cm at 4 weeks post-operation and were significantly different, 1.80 vs. 1.20 cm and 1.88 vs. 1.28 cm. These data objectively indicate the fluctuations of the hip and knee joints during walking in SNL rats. A previous paper reported that toe and ankle angles were decreased in SNL rats in a 3D kinematic analysis [25]; however, there were no data on vertical fluctuations in each joint correlating with walking characteristics in patients with neuropathic pain.

Second, we revealed that heel step length and both-foot contact time were significantly longer on the left compared to the right side in the SNL rats. Third, in the circular phase, the *R* values were also decreased in the SNL rats. The ratio of the right/left circular time was 0.62 ± 0.15 in the SNL rats. These data indicate that the walking patterns of the left and right lower limbs in SNL rats are asymmetrically different from those of the sham rats. Some papers measured step length and both-foot contact time in a 2D catwalk system and obtained results similar to ours [9, 12]. However, a report by Kanchiku et al. was the first report to reveal asymmetrical walking disability by calculating the *R* values in the circular phase [17].

There are some limitations to the collection of accurate data with 3D kinematic gait analysis. Researchers need to train rodents to walk on the treadmill smoothly, to attach markers correctly to each joint, and to train themselves in the tracing examination processes [21]. Furthermore, the high cost of 3D kinematic analysis equipment may impede its popularization and limit its use in relevant studies. However, this new analysis system for SNL and neuropathic pain in an experimental model could contribute to the development of new treatments for patients with sciatic nerve damage and neuropathic pain because we can evaluate the rodent model using clinically relevant methods that have never been available before.

## Conclusions

We revealed new quantitative parameters in an SNL rat model that are directly relevant to neurological symptoms in patients with neuropathic pain. Sagittal trajectories of the hip and knee markers in SNL rats showed a large sagittal fluctuation of each joint while walking. Top minus bottom height of the left hip and knee that represents instability during walking was significantly larger in the SNL than sham rats. Both-foot contact time was significantly longer in the SNL rats. Circular phase time was used to evaluate the coordination of right and left hind-limbs. The ratio of the right/left circular time was 0.62 ± 0.15 in the SNL rats. This new clinically relevant evaluation method in a rodent model can generate in vivo pain research data that is more reflective of clinical morbid conditions than other conventional methods.

## Methods

Six-week-old male Sprague-Dawley rats weighing about 200 g each were used in this study (Chiyoda Kaihatsu Co., Tokyo, Japan). Animal experiments were carried out in accordance with the Guidelines for Animal Experimentation of the Yamaguchi University School of Medicine and conformed to Animal Research: Reporting in Vivo Experiments (ARRIVE) guidelines. The protocols were approved by the Institutional Animal Care and Use Committee of Yamaguchi University (15-044). The animals were given water and food ad libitum and housed in a climate-controlled room. They were maintained under reverse light–dark conditions from 8 pm to 8 am [7]. All efforts were made to minimize suffering. All rats received an analgesic (carprofen, 5 mg/kg, subcutaneous) immediately before and the next day after surgery. Each rat was monitored until awake and moving freely around the recovery chamber. Animals were then single-housed for the duration of the study. The methods of euthanasia consisted of an overdose of sodium pentobarbital injected intraperitoneally (> 100 mg/kg).

### Spinal nerve ligation

**Fig. 7:**
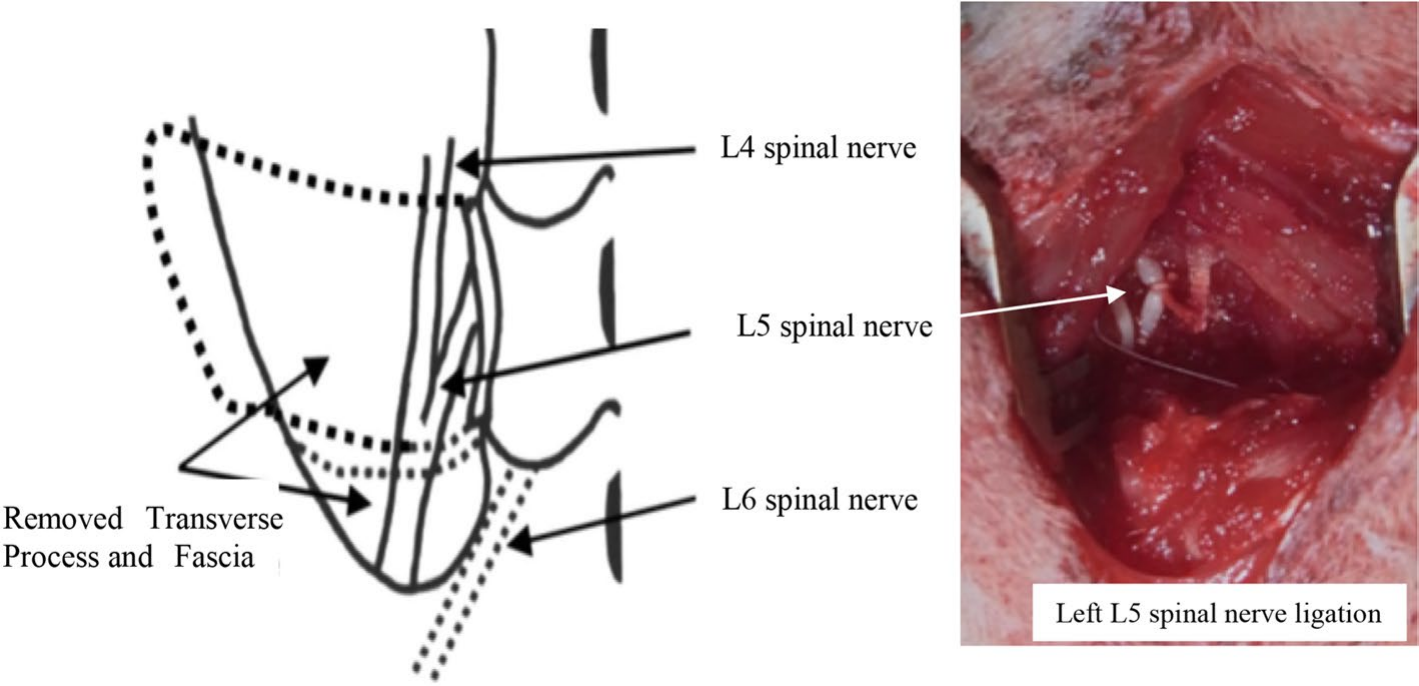
Performing sciatic nerve ligation of the left L5 spinal nerve. Left panel: schematic of the procedure in which the L5 spinal nerves were isolated and tightly ligated with 6–0 silk sutures distal to the dorsal root ganglion (SNL = 10). Right panel: operative photograph

L5 SNL (*n* = 10 rats) was performed according to the method devised by Kim and Chung (Fig. 7) [4, 5]. The paraspinal muscles at the vertebrae level of the left fourth lumbar spinal nerve (L4) to the second sacral spinal nerve (S2) were separated from the vertebral spinous processes, and the left L6 transverse process was removed to expose the L4–L5 spinal nerves. The left L5 spinal nerves were isolated and tightly ligated with 6–0 silk sutures distal to the dorsal root ganglion (Fig. 7). The incision was sutured with 4–0 nylon. Sham operated animals (*n* = 10) were prepared in an identical manner but without ligation of the spinal nerves. Rats with sciatic nerve injury, as judged by an inverted foot with ventro-flexed toes, were removed from the study [4, 5].

### Assessment of tactile sensitivity

Animals were examined after 7 days of habituation to the environment. Decreases in the mechanical withdrawal threshold were interpreted as hyperalgesia [2, 6, 8]. Behavioral tests were conducted one day before surgery and then periodically after surgery. Rats were allowed to acclimatize for 15 min within acrylic glass enclosures. Tactile allodynia of the plantar surface was assessed using an automated von Frey Dynamic Plantar Aesthesiometer (Ugo Basile, Comerio, Italy) [7]. The force was initially below the detection threshold and was then increased from 1 to 50 g over 20 s [7, 8]. The withdrawal response evoked by mechanical stimulation was determined by the extent of foot lifting and shaking. Mechanical stimulation was repeated 3 times at intervals of 5 min. The average result from 3 tests was used to determine the mechanical allodynia [6–8].

### Three-dimensional kinematic analysis

**Fig. 8:**
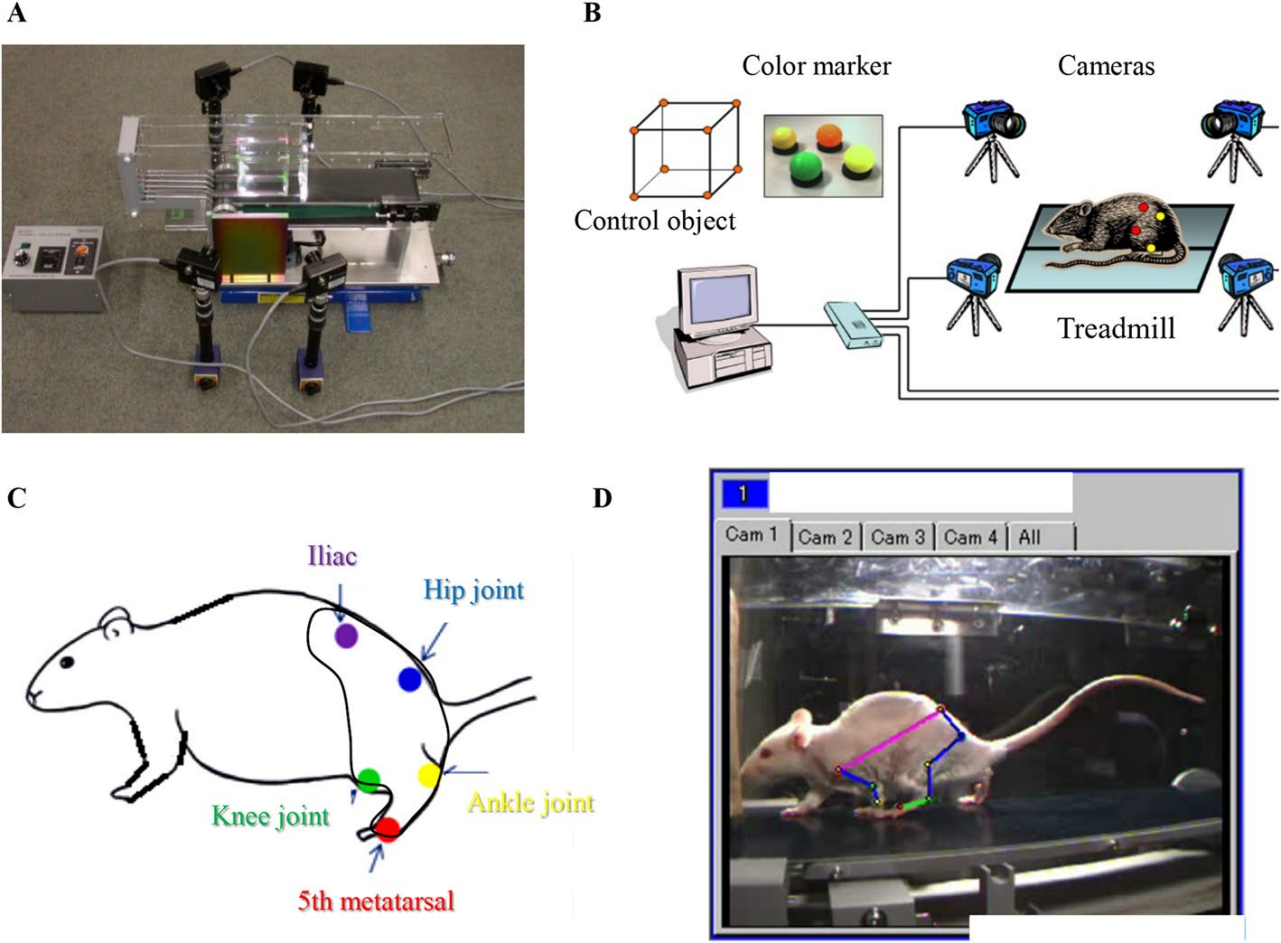
3D Digital gait analysis. **A**, **B** Images and schematic of the four-camera kinematic data collection set-up for a treadmill walking task. **B** Four cameras are placed with two on either side of the treadmill to capture (frame-by-frame) left and right marker motions during locomotion. **C** Diagram depicting marker placement over bony landmarks on the rodent’s hind-limbs to capture locomotion kinematics. In total, five markers are placed on each side of the rat. The shaded regions show the area where the rat is shaved. **D** Video capture of 3D kinematics

Hind-limb movement was assessed using the Kinema-Tracer system (Kissei Comtec) (Fig. 8A–D) [16, 17]. Under light anesthesia with sevoflurane, colored markers were attached to the skin at the iliac region, hip, knee, ankle and 5th metacarpophalangeal joints with glue as in a previously reported method (Fig. 8C) [16, 17]. Four cameras (Point Grey Research, Richmond, Canada) were used to film the markers (Fig. 8B). Before each session, the precise coordinates were calibrated by recording a cube of known size (5 × 20 × 10 cm [*x* × *y* × *z*]) [16, 17]. Two weeks prior to the experiments, rats were trained to walk on a treadmill for 20 min at 12 m/min. Sham (*n* = 10) and SNL (n = 10) rats were analyzed one day before surgery and at 7 and 28 days after surgery. Each session involved several trials so that rats performed successively. Data from a total of 10 steps were obtained for each animal. The position of the joint in the *xyz* plane was automatically calculated. The timing of foot contact and lift-off was manually entered into the Kinema-Tracer (Fig. 8D) [16, 17]. We compared the trajectories of the hip, knee and ankle joints before and after surgery in the SNL rats. Stride length was defined as the phase distance between contacts by the same limb, and step length as the distance between contacts by one limb and the contralateral limb (Additional file 1: Fig S1 and Fig. 5). We also compared the average stride and step lengths, both-foot contact time and circular phase time between the sham and SNL models [16, 17].

Quantitative assessment of interlimb gait coordination was conducted to determine the consistency of 1:1 correspondence between right and left hind-limbs and to establish the relative phase of each limb touchdown with respect to another within a gait cycle. In this study, mean and *R* values of the ratio of left/right circular time were calculated in the circular phase to assess the consistency of 1:1 hind-limb right-left interlimb coordination [15–17]. The mean *R* values were normalized to 1.

### Statistical analysis

Data are presented as mean ± standard error (SE) and were analyzed using StatFlex Ver. 7 for Windows (Artec, Osaka, Japan; https://www.statflex.net/). For von Frey test analysis (Fig. 1), comparisons of withdrawal threshold times between left and right feet at each time point were made by use of a paired t-test. For 3D kinematic analysis (Figs. 2, 3, 4 and 5), comparisons of results between the SNL and sham groups were performed by Mann–Whitney test, whereas left to right comparisons of values within SNL rats were performed by paired *t*-test. A *p*-value of less than 0.05 was deemed statistically significant.

## Supplementary Information

**Additional file 1: Figure S1.** Figure Walking phases of the stance and swing phase, step length and stride length.

## Data Availability

The datasets used and/or analyzed during the current study are available from the corresponding author on reasonable request.
