# Supplementary figures and images for: Three-dimensional analysis of the characteristics of joint motion and gait pattern in a rodent model following spinal nerve ligation
Source: Biomed Eng Online. 2021 Jun 5;20:55. doi: 10.1186/s12938-021-00892-6 (PMC8180104; doi:10.1186/s12938-021-00892-6)

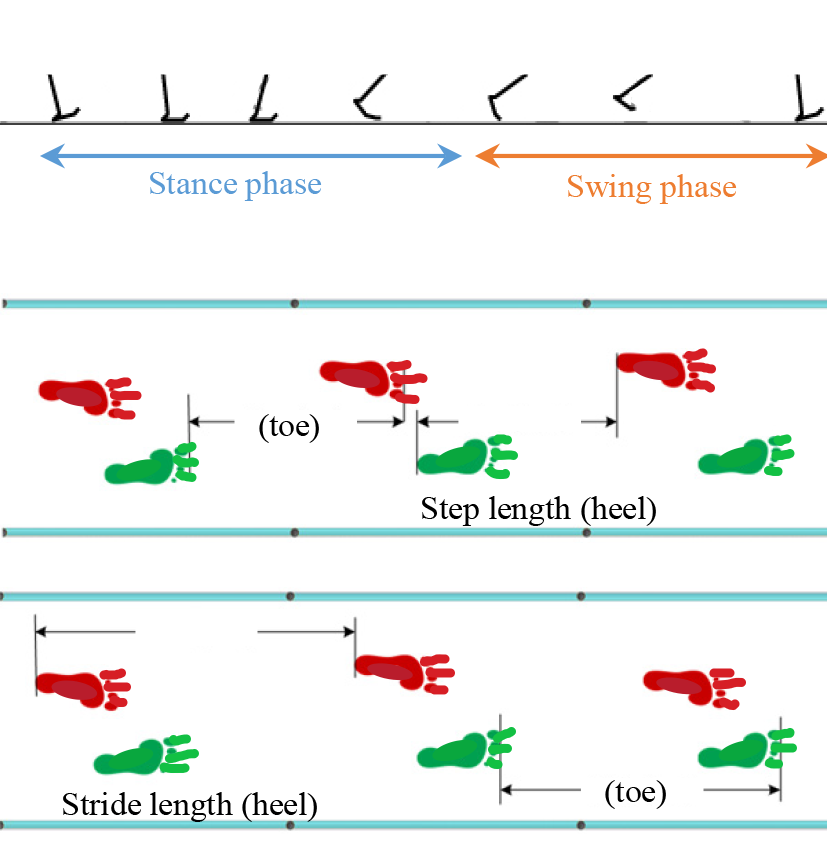

Supplement: Supplementary file 1 — Additional file 1: Figure S1. Figure Walking phases of the stance and swing phase, step length and stride length. [file 12938_2021_892_MOESM1_ESM.tif]
